# The Occurrence of Richter's Syndrome during Treatment with Obinutuzumab and Chlorambucil

**DOI:** 10.1155/2020/8363427

**Published:** 2020-07-16

**Authors:** Teng Fong Ng, Benedict Carnley, Celia Green, Dominic Spagnolo, Michael F. Leahy

**Affiliations:** ^1^Royal Perth Hospital, Perth, WA, Australia; ^2^PathWest Laboratory Medicine, Perth, WA, Australia

## Abstract

Chronic lymphocytic leukaemia is a slow-growing leukaemia of developing B-lymphocytes, which may transform to an aggressive lymphoma known as Richter's syndrome. While Richter's syndrome can present in untreated or relapsed-refractory cases, it may occur upon the commencement of less intensity treatment regimens. We present a case of Richter's syndrome following treatment with chlorambucil and obinutuzumab and review of available literature on the topic.

## 1. Background

Richter's syndrome (RS) is the development of an aggressive large cell lymphoma in the setting of the chronic lymphocytic leukaemia (CLL) or small lymphocytic leukaemia (SLL). RS was first described by Maurice Richter in 1928 as a “generalized reticular cell sarcoma” in a patient with CLL [1]. Clinically, RS is characterised by sudden clinical deterioration, marked increase in lymphadenopathy or splenomegaly, worsening “B” symptoms (i.e., fever, night sweats, weight loss), and increased serum lactate dehydrogenase (LDH) levels. RS occurs in approximately 2%–10% of CLL patients during the course of their disease [2–7]. The most common transformation is diffuse large B-cell lymphoma (DLBCL), but occasionally, CLL may transform into Hodgkin lymphoma [8]. Because of its dismal prognosis and aggressive nature, RS requires intensive chemoimmunotherapy with regimens such as R-CHOP and consolidation with haemopoietic stem cell transplantation.

## 2. Case Presentation

A 47-year-old Anglo-Saxon lady with chronic lymphocytic leukaemia, with weight loss, night sweats, and gradually progressive global lymphadenopathy, was commenced on treatment with a second-generation monoclonal anti-CD20 antibody obinutuzumab together with chlorambucil. The reduced intensity treatment is chosen as she has significant comorbidities which include ventricular septal defect, obesity, diabetes mellitus, chronic bronchitis, osteoarthritis limiting mobility, depression, recurrent migraine, and possibly tracheomalacia. Her pretreatment complete full blood count showed haemoglobin level of 158 g/L, elevated white cell count of 76 × 10^9^/L with lymphocyte count of 68 × 10^9^/L, and normal platelet count of 260 × 10^9^/L. Serum lactate dehydrogenase was 280 U/L. The immunophenotype of her CLL is CD5+, CD19+, CD20+, CD23+, and CD200+, with lambda clonal restriction. Previous cytogenetic analysis was unremarkable, but molecular genetic analysis was not undertaken.

**Figure 1:**
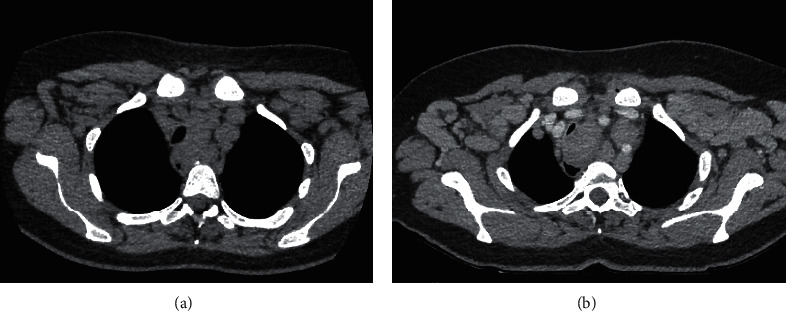
Computed tomography of the chest showing the rapidly expanding mediastinal lymphadenopathy. The trachea was compressed with the narrow diameter of <1 cm. (a) Pretreatment. (b) Day 14 following commencement of treatment.

Cycle 1 of the 28-day protocol consisted of chlorambucil 0.5 mg/kg on day 1 and day 15, together with obinutuzumab 1000 mg on day 1-2 in split doses, day 8, and day 15. Subsequent 5 cycles have similar dosing of chlorambucil, but only one dose of obinutuzumab 1000 mg on day 1 is given [9]. On day 13 of cycle 1, 5 days after receiving obinutuzumab, she presented with dyspnoea, stridor, dysphagia, and increasing cervical lymphadenopathy. An urgent computed tomography scan demonstrated cervical and mediastinal lymphadenopathy resulting in tracheal compression (see Figure 1). Her serum LDH had increased from pretreatment levels to 1260 U/L, but other tumour lysis markers remained unremarkable. Her peripheral blood lymphocyte count had declined to 0.3 × 10^9^/L.

High dose intravenous dexamethasone was promptly commenced to attempt to reduce lymphadenopathy to alleviate the impending clinical airway compromise. In addition, the patient was empirically treated with antiherpetic treatment with intravenous high dose acyclovir as she had persistent herpes simplex labialis. This was due to concern of rapidly progressing herpetic lymphadenitis as a key differential diagnosis because there are case reports that has described similar presentation occurring during anti-CLL treatment [10, 11].

**Figure 2:**
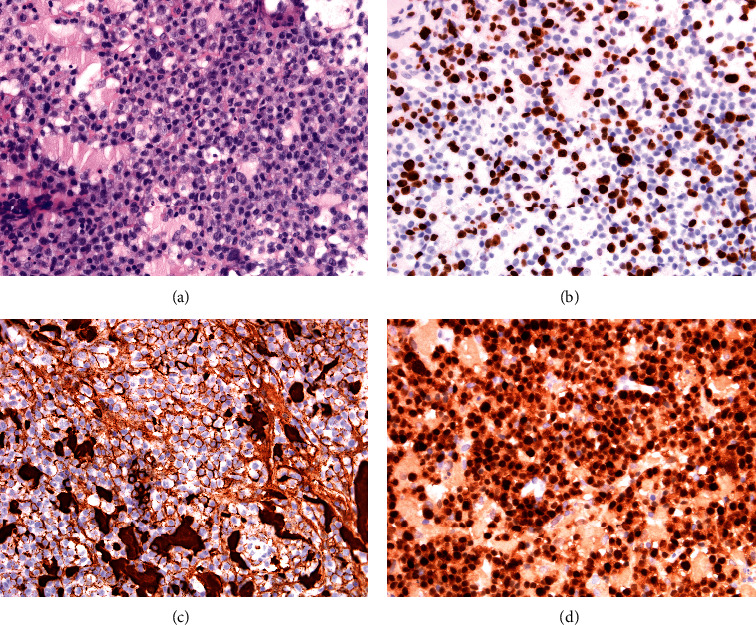
Sheets of large lymphocytes with centroblastic morphology ((a) haematoxylin and eosin stain), with positive immunohistochemistry for high Ki-67 index of 70%, CD20, and MUM1 (b)–(d), respectively.

A percutaneous cervical lymph node biopsy was performed to obtained specimen for laboratorial analysis. Histologically, there was infiltration of skeletal muscle (there was no nodal tissue) by a confluent and sheet-like growth of centroblastoid cells accompanied by coagulative necrosis. The cells had large round to ovoid vesicular nuclei, one or more prominent nucleoli, often located on the nuclear membrane, and ample amphophilic or cleared cytoplasm. Mitoses were evident in any single high-power field and spotty apoptosis was present throughout. Based on paraffin section immunohistochemistry, the neoplastic cells were positive for CD20, CD5 (weak), CD23, CD43, BCL2 (>70%), and MUM1 and were lambda light-chain restricted; the Ki-67 proliferation index was approximately 70% (See Figure 2). The lymphoma was negative for CD10, CD30, cyclin D1, SOX11, C-MYC (<10% staining), BCL6, CD138, kappa light chain, HSV1/2, and EBER (by in situ hybridization). Interphase fluorescence in situ hybridization (FISH) studies on paraffin sections confirmed the presence of an IgH disruption but there was no disruption of BCL6 or MYC. Based on the Hans classifier, the features were those of an activated B-cell-like phenotype, with retained expression of the parent CLL markers CD5 (weak) and CD23, and not those of a double-expressor DLBCL (BCL2 high, MYC low). These large cells also exhibited similar lambda light-chain restriction which may imply a clonal relationship. Cytogenetic analysis did not identify ATM (11q22) or TP53 (17p13) deletions.

Following the histological diagnosis of RS, the patient was commenced on R-CHOP chemotherapy regimen, which resulted in reduction of lymphadenopathy and resolution of stridor and dyspnoea. Upon completion of 6 cycles, the patient achieved complete metabolic remission by a position emission tomography (PET) scan.

## 3. Discussion

RS may occur in CLL patients during the “watchful waiting” period before requiring any CLL treatment and can be a late event occurring following CLL treatment [5]. The incidence of RS increases with the number of prior therapies [12, 13]. Approximately, 50% cases of RS occur before the treatment of CLL [7]. However, there is little detail in reports in the literature about when during therapy. Most reports give a range that can start from 0 months which implied that RS occurs during the early period of treatment [7, 14].

Chlorambucil has been a long-standing alkylating agent used in the treatment of CLL since 1970s [15]. The risk of RS with treatment with chlorambucil alone is about 5%, observed in the prospective CALGB 9011 trial [16]. However, there is limited report of RS being precipitated by treatment with chlorambucil alone in the literature, which may be because the phenomenon is not highlighted in the relevant publications.

Recently, RS has been described during novel targeted treatments, notably the BCL2 inhibitor venetoclax [17]. Many of the RS cases developed shortly after starting treatment, suggesting unrecognised RS predates the initiation of the novel targeted therapy [18]. In the phase-1 venetoclax trial in heavily treated relapse-refractory CLL, 16% of patients on venetoclax develop RS and considered that as treatment failure and progressive disease [19]. The authors reported that RS appears to represent a mechanism of tumour escape from suppression by the inhibition of BCL2, particularly for patients with CLL with 17p deletion. RS was also observed in 8 of 25 patients with disease progression who were receiving ibrutinib monotherapy in an extended follow-up study [20].

There are some reports of the occurrence of RS in major clinical trials of treatment approaches using anti-CD20 monoclonal antibodies, but these rates are not well highlighted. In the CLL8 phase-3 trial investigating the efficacy of fludarabine and cyclophosphamide (FC) with 1^st^ generation monoclonal anti-CD20 chimeric antibody rituximab, 13 out of 408 (3%) patients on the FC-rituximab treatment arm developed RS on long-term follow-up [21]. In the iLLUMUNATE trial, there were 2 out of 116 patients (2%) in the control arm treated with chlorambucil and obinutuzumab that developed RS as progression of disease [22]. In the CLL-BAG02 trial, 3 out of 66 patients (4.5%) experienced RS following treatment with bendamustine and obinutuzumab regimen [23]. The early trial of single agent ofatumumab had 1 case of RS in a patient that has been heavily treated with fludarabine and alemtuzumab [15]. In the phase-2 trial using bendamustine and ofatumumab in relapsed CLL patients, 3 out of 10 patients developed RS [16]. Of interest, 2 of these patients developed RS around cycle 2 of the treatment.

In our case, the chlorambucil and obinutuzumab combination seemed to have rapidly precipitated RS after the doses of both agents in a treatment naïve patient. This is similar timing to the 2 patients reported in the phase-2 bendamustine and ofatumumab trial [16]. In these cases, it is possible that the aggressive large cell clone may be present initially as a minor clone [24], and thus these patients do not exhibit the full clinical manifestations of RS until the commencement of the effective anti-CLL treatment. Our patient has limited clinical manifestations of RS including night sweats and weight loss, but pretreatment of serum lactate dehydrogenase is not markedly elevated till after the 2^nd^ infusion of the anti-CD20 monoclonal antibody.

There is a possibility that the RS clone was clonally selected due to rapid cytoreduction of the treatment-sensitive CLL clones by chlorambucil and obinutuzumab. However, we do not have molecular studies to ascertain the origin of the RS in our case to determine whether it is clonally related or clonally unrelated. Rossi et al. have pointed out that the clonally related RS stems from a subclone cell that is present CLL initially, which become the dominant subclone during the clinical RS transformation as other CLL subclones reduce in proportion [25], whereas clonal unrelated RS is biologically different, characterised by a significantly higher mutation frequency. Interestingly, patients with clonally unrelated RS have a median survival of approximately 62 months, which is comparable to patients with de novo DLBCL [6]. On the contrary, the median survival of patients with clonally related RS is significantly shorter, ranging from 8 to 14 months [6]. Clonal dynamics monitoring of clonal unrelated RS has demonstrated clonal evolution, acquiring and selecting new mutations in response to treatment, and loss of cellular tumoral clone during progression [26].

In conclusion, this case spotlighted an uncommon phenomenon in CLL where RS can rapidly present following the commencement of the anti-CLL regimen. RS requires rapid histological confirmation and prompt treatment with intensive immunochemotherapy for large cell lymphoma in order to achieve a clinical response. We hope that this phenomenon can be reported distinctly in the future publications regarding RS.

## References

[1] Richter M. N. (1928). Generalized reticular cell sarcoma of lymph nodes associated with lymphatic leukemia. *The American Journal of Pathology*.

[2] Mauro F. R., Foa R., Giannarelli D. (1999). Clinical characteristics and outcome of young chronic lymphocytic leukemia patients: a single institution study of 204 cases. *Blood*.

[3] Thornton P. D., Bellas C., Santon A. (2005). Richter’s transformation of chronic lymphocytic leukemia: the possible role of fludarabine and the epstein-barr virus in its pathogenesis. *Leukemia Research*.

[4] Tsimberidou A.-M., Keating M. J. (2005). Richter syndrome: biology, incidence, and therapeutic strategies. *Cancer*.

[5] Maddocks-Christianson K., Slager S. L., Zent C. S. (2007). Risk factors for development of a second lymphoid malignancy in patients with chronic lymphocytic leukaemia. *British Journal of Haematology*.

[6] Rossi D., Spina V., Deambrogi C. (2011). The genetics of Richter syndrome reveals disease heterogeneity and predicts survival after transformation. *Blood*.

[7] Parikh S. A., Rabe K. G., Call T. G. (2013). Diffuse large B-cell lymphoma (Richter syndrome) in patients with chronic lymphocytic leukaemia (CLL): a cohort study of newly diagnosed patients. *British Journal of Haematology*.

[8] Campo E., Ghia P., Montserrat E., Swerdlow S. H., Campo E., Harris N. L. (2017). Chronic lymphocytic leukaemia/small lymphocytic lymphoma. *WHO Classification of Tumours of Haematopoietic and Lymphoid Tissues*.

[9] Goede V., Fischer K., Busch R. (2014). Obinutuzumab plus chlorambucil in patients with CLL and coexisting conditions. *New England Journal of Medicine*.

[10] Villa D., Skinnider B. (2014). Herpes simplex virus lymphadenitis. *Blood*.

[11] Srivastava R., Griswold D., Jamil M. O. (2018). Chronic lymphocytic leukaemia with necrotic herpetic adenitis: an elusive clinical condition. *BMJ Case Reports*.

[12] Robertson L. E., Pugh W., O’Brien S. (1993). Richter’s syndrome: a report on 39 patients. *Journal of Clinical Oncology*.

[13] Tsimberidou A. M., O’Brien S., Khouri I. (2006). Clinical outcomes and prognostic factors in patients with Richter’s syndrome treated with chemotherapy or chemoimmunotherapy with or without stem-cell transplantation. *Journal of Clinical Oncology*.

[14] Fan L., Wang L., Zhang R. (2012). Richter transformation in 16 of 149 Chinese patients with chronic lymphocytic leukemia. *Leukemia & Lymphoma*.

[15] Wierda W. G., Kipps T. J., Mayer J. (2010). Ofatumumab as single-agent CD20 immunotherapy in fludarabine-refractory chronic lymphocytic leukemia. *Journal of Clinical Oncology*.

[16] Ujjani C., Ramzi P., Gehan E., Wang H., Wang Y., Cheson B. D. (2015). Ofatumumab and bendamustine in previously treated chronic lymphocytic leukemia and small lymphocytic lymphoma. *Leukemia & Lymphoma*.

[17] Anderson M. A., Tam C., Lew T. E. (2017). Clinicopathological features and outcomes of progression of CLL on the BCL2 inhibitor venetoclax. *Blood*.

[18] Parikh S. A., Kay N. E., Shanafelt T. D. (2014). How we treat Richter syndrome. *Blood*.

[19] Roberts A. W., Davids M. S., Pagel J. M. (2016). Targeting BCL2 with venetoclax in relapsed chronic lymphocytic leukemia. *New England Journal of Medicine*.

[20] Byrd J. C., Furman R. R., Coutre S. E. (2015). Three-year follow-up of treatment-naïve and previously treated patients with CLL and SLL receiving single-agent ibrutinib. *Blood*.

[21] Fischer K., Bahlo J., Fink A.-M. (2012). Extended follow up of the CLL8 protocol, a randomized phase-III trial of the German CLL study group (GCLLSG) comparing fludarabine and cyclophosphamide (FC) to FC plus rituximab (FCR) for previously untreated patients with chronic lymphocytic leukemia (CLL): results on survival, progression-free survival, delayed neutropenias and secondary malignancies confirm superiority of the FCR regimen. *Blood*.

[22] Moreno C., Greil R., Demirkan F. (2019). Ibrutinib plus obinutuzumab versus chlorambucil plus obinutuzumab in first-line treatment of chronic lymphocytic leukaemia (iLLUMINATE): a multicentre, randomised, open-label, phase 3 trial. *The Lancet Oncology*.

[23] Cramer P., Tresckow J. V., Bahlo J. (2018). Bendamustine followed by obinutuzumab and venetoclax in chronic lymphocytic leukaemia (CLL2-BAG): primary endpoint analysis of a multicentre, open-label, phase 2 trial. *The Lancet Oncology*.

[24] Timár B., Fülöp Z., Csernus B. (2004). Relationship between the mutational status of VH genes and pathogenesis of diffuse large B-cell lymphoma in Richter’s syndrome. *Leukemia*.

[25] Rossi D., Spina V., Forconi F. (2012). Molecular history of Richter syndrome: origin from a cell already present at the time of chronic lymphocytic leukemia diagnosis. *International Journal of Cancer*.

[26] González-Rincón J., Gómez S., Martinez N. (2019). Clonal dynamics monitoring during clinical evolution in chronic lymphocytic leukaemia. *Scientific Reports*.

